# Upper Tract Urothelial Carcinoma: A Rare Malignancy with Distinct Immuno-Genomic Features in the Era of Precision-Based Therapies

**DOI:** 10.3390/biomedicines11071775

**Published:** 2023-06-21

**Authors:** Konstantinos Evmorfopoulos, Lampros Mitrakas, Athanasios Karathanasis, Ioannis Zachos, Vassilios Tzortzis, Panagiotis J. Vlachostergios

**Affiliations:** 1 Department of Urology, School of Health Sciences, Faculty of Medicine, University of Thessaly, 41110 Larissa, Greece; 2 Department of Medical Oncology, IASO Thessalias Hospital, 41500 Larissa, Greece; 3 Division of Hematology and Medical Oncology, Department of Medicine, Weill Cornell Medicine, New York, NY 10065, USA

**Keywords:** upper tract urothelial carcinoma, cisplatin-based chemotherapy, FGFR3, luminal papillary, T-cell depleted, immune checkpoint inhibitors, lynch syndrome, radical nephroureterectomy

## Abstract

Upper tract urothelial carcinoma (UTUC) is a rare malignancy, occurring in 5–10% of patients diagnosed with UC, and involves the renal pelvis, calyces, or ureters. UTUC can be sporadic or hereditary as a clinical manifestation of Lynch syndrome. Therapeutic management of these patients is challenging. Following risk stratification of localized disease, patients with low-grade UTUC may undergo kidney-sparing surgery or radical nephroureterectomy (RNU) and/or chemoablation with mitomycin-c instillation to reduce recurrence. In high-grade disease, RNU followed by adjuvant chemotherapy remains the standard of care. For decades, platinum-based chemotherapy has been the cornerstone of treatment for locally advanced and metastatic disease. The aim of the present review is to summarize recent advances in UTUC’s therapeutic management through the lens of its genomic and immune landscape. Accumulating knowledge on the genetic and immune aspects of UTUC tumors has increased our understanding of their underlying biology, supporting a luminal papillary, T-cell depleted contexture and enrichment in fibroblast growth factor receptor (FGFR) expression. These advances have fueled successful clinical testing of several precision-based therapeutic approaches, including immune checkpoint inhibitors (ICIs), the antibody–drug conjugates (ADCs) enfortumab vedotin and sacituzumab govitecan, and agents targeting the FGFR axis such as erdafitinib and other kinase inhibitors, allowing their entry into the therapeutic armamentarium and improving the prognosis of these patients. Not all patients respond to these precision-based targeted therapies; thus, validating and expanding the toolkit of potential biomarkers of response or resistance, including molecular subtypes, FGFR pathway gene alterations, DNA repair gene defects, tumor mutational burden (TMB), circulating tumor DNA (ctDNA), nectin-4, TROP2, and programmed death ligand-1 (PD-L1), are key to maximizing the benefit to these particular subgroups of patients.

## 1. Introduction

Upper tract urothelial carcinoma (UTUC) accounts for approximately 5–10% of cases diagnosed with urothelial carcinoma (UC) [1]. While UTUC presents in pyelocaliceal cavities and ureters, approximately half of UTUC cases will exhibit concurrent UC of the bladder (UCB) [1]. In Western countries, UTUC has an incidence of 1–2 cases per 100,000, diagnosed mainly in older patients; in some Asian countries, UTUC accounts for 25% of all UC cases, presenting with more aggressive features [2,3]. UTUC and UCB share common risk factors, including cigarette smoking, occupational exposure, and a high dietary intake of aristolochic acid. UTUC may also occur as part of Lynch syndrome, also known as hereditary non-polyposis colorectal cancer syndrome, due to germline mutation in DNA mismatch repair genes [4,5]. Nevertheless, the majority (80%) of UTUCs are not associated with LS but emerge as a result of sporadic events. UTUC may present with aggressive clinical behavior at diagnosis. More than half of patients are found to have muscle-invasive disease, which is often already metastatic [6]. Moreover, UTUC is also associated with poor clinical outcomes demonstrating a 5-year specific survival of less than 50% and 10% for pT2/T3 and pT4 tumor stages, respectively [7]. Radical nephroureterectomy (RNU) with lymphadenectomy and bladder cuff removal is the standard of care for these patients, while the addition of perioperative platinum-based combination therapy has led to an improvement in prognosis compared to RNU alone and constitutes the gold standard of treatment according to international guidelines [8,9,10]. In the metastatic setting, platinum-based chemotherapy remains the initial treatment of choice in eligible patients. The emergence of immune checkpoint inhibition (ICI), particularly against the programmed death-1 (PD-1)/PD-ligand 1 (PD-L1) axis, as a therapeutic strategy for maintenance or beyond progression to platinum-based chemotherapy has offered a significant clinical benefit to UC patients, including those with upper tract disease [8,9,10]. Additionally, recent studies have revealed an important role of FGFR-, nectin-4-, and TROP2-targeting with tyrosine kinase inhibitors and antibody–drug conjugates (ADCs), respectively, in further improving the prognosis of UC patients with platinum- and/or ICI-refractory disease [11,12].

All of these advances in the therapeutic armamentarium of UTUC would have been impossible without molecular characterization studies in UC. In the era of precision medicine, the entry of novel technologies including next-generation sequencing (NGS) in cancer diagnosis and treatment has transformed our knowledge in predicting the activity of targeted therapies exploiting the defects or specific oncogene addiction present in various tumor types. In UTUC, an increasing number of studies have focused on the molecular characterization of the disease in an effort to understand the underlying biology of this tumor and identify potential therapeutic targets and corresponding biomarkers or response and resistance. Despite UTUC being poorly represented in the Cancer Genome Atlas (TCGA) data, smaller studies focusing exclusively on UTUC have revealed distinct molecular and genetic features of UTUC compared to UCB [13]. Elucidation of the genomic and immune landscape of UTUC has played an important role in understanding the key pathways involved in its development and progression, resulting in an explosion of novel targeted therapies and combinations [13]. The aim of this review is to provide a comprehensive synopsis of all recent studies focusing on the clinical and molecular aspects of the disease at the diagnostic and therapeutic levels, as well as to provide an insight into potential surrogate markers of response and resistance that could be used to guide therapeutic decisions in specific subsets of the disease.

## 2. Diagnosis and Staging of UTUC

The diagnosis and staging of UTUC involves a combination of urine cytology, imaging studies, and endoscopy in order to stratify patients into risk groups for tailoring treatment selection.

Computed tomographic (CT) urography is an integral part of the diagnosis and staging of these patients, with a sensitivity and specificity of 92% and 95%, respectively [14]. However, radiation exposure, nephrotoxicity, and allergic reactions after iodine contrast administration must be taken under consideration. When CT urography is contraindicated, magnetic resonance urography represents an alternative imaging study. However, it lacks sensitivity (around 75%), compared to CT urography, in diagnosing tumors < 2 cm [15]. In the metastatic setting, ^18^F-fluorodeoxyglucose positron emission tomography/computed tomography (FDG-PET/CT) can be performed for the detection of nodal metastases, demonstrating a sensitivity and specificity of 82 and 84%, respectively [16]. Patients with suspicious lymph nodes on FDG-PET/CT experience a worse recurrence-free survival [16].

Endoscopy with ureteroscopy (URS) and cystoscopy remains the cornerstone in the diagnosis of patients with UTUC. Direct visualization of a suspected lesion in the ureter or the pyelocaliceal system using a flexible or rigid ureteroscope is used to confirm the diagnosis and assess its main features, including appearance, multifocality, and size, stratifying patients into low- and high-risk groups [1]. Selective ureteral sampling for cytology in situ can also be obtained [17,18], while performing a biopsy of the suspected lesion is questionable due to the observed under-staging occurring with URS as compared to RNU [19,20]. Additionally, in a meta-analysis comparing the use of URS prior to RNU, 8 out of 12 studies found that there was a higher risk of intravesical recurrence if URS was performed before RNU compared to conducting RNU without prior URS [21]. Conducting a biopsy during URS was also identified as a factor increasing the risk of intravesical recurrence [21]. Another systematic review of 16 studies indicated that URS alone did not have a significant association with intravesical recurrence; in contrast, URS combined with a biopsy significantly increased the risk of subsequent intravesical recurrence, although it did not have an independent effect on long-term survival outcomes [22]. Further to diagnostic URS, urethrocystoscopy is also important in the diagnostic workup of UTUC due to the presence of concomitant UCB in around 50% of patients [1].

Urinary cytology can also be used in the diagnosis and staging of UTUC patients. Abnormal cytology indicates high-grade disease when cystoscopy is normal, and there is no evidence of carcinoma in situ in the bladder and prostatic urethra [23]. However, cytology is less sensitive in the diagnosis of UTUC compared to UCB and should be performed selectively from the affected upper tract [24].

## 3. Clinical Phenotype and Management of Localized UTUC

According to the European Association of Urology (EAU), UTUC can be stratified into low-risk and high-risk disease based on clinical, endoscopic, radiographic, and histopathologic factors [1,25]. Low-risk localized disease is unifocal—with a tumor size < 2 cm, low-grade cytology, and/or URS biopsy—and noninvasive on imaging. In contrast, high-risk disease is characterized by hydronephrosis, multifocal tumors > 2 cm, high-grade cytology and/or URS biopsy, invasive disease on imaging, and a history of prior UCB treated with radical cystectomy [1,25].

Patients presenting with low-risk localized disease can be treated with kidney-sparing surgical approaches including segmental ureterectomy, ureteroscopy, percutaneous tumor resection, or radical nephroureterectomy (RNU). Kidney-sparing approaches reduce the morbidity associated with radical surgery and are well-suited for patients possessing a solitary kidney or compromised renal function [1,25]. However, offering kidney-sparing surgery must be accompanied by meticulous and strict follow-up with repeat cystoscopy, ureteroscopy, upper urinary tract imaging, and urine cytology to avoid compromising oncological outcomes [26].

In order to reduce recurrence rates in patients undergoing kidney-sparing surgeries, several studies have examined the use of intraluminal therapies with Bacillus Calmette–Guérin (BCG) or mitomycin-c (MMC) in low-risk localized UTUC. Instillation of these agents in the renal pelvis or the ureter can be performed with a percutaneous approach through a nephrostomy catheter or retrograde through a single J open-end ureteric stent, while the use of a double J stent is not recommended because the reflux may be inadequate to transfer the drug to the renal pelvis [27]. However, both percutaneous and retrograde administration of these drugs may be complicated by ureteric obstruction and pyelovenous influx. Several studies have evaluated recurrence rates after administration of BCG in the adjuvant setting of low-risk localized UTUC [27,28,29] or as a primary therapy for carcinoma in situ [27,28,30,31]. These studies have reported a recurrence rate of 13–59% after adjuvant therapy with BCG. However, administration of adjuvant BCG after endoscopic tumor resection did not result in significantly lower recurrence rates (26% for endoscopic management alone and 33% for those receiving adjuvant BCG) [29]. Similar results were reported in a meta-analysis by Foerster et al. [32]. An insufficient concentration of the drug for an adequate time period, due to urine excretion, seems to be the main reason why BCG instillation has been an inadequate measure for preventing recurrences.

Mitomycin-c is another drug that has been studied for intraluminal treatment of low-grade UTUC. Recently, UGN-101 (JELMYTO), a mitomycin-containing reverse thermal gel (4 mg MMC/mL) was evaluated in the OLYMPUS study, a phase-III trial, showing promising results after kidney-sparing surgery [33]. UGN-101 exhibits a liquid form in a cold environment. Upon instillation, it becomes a semisolid gel at body temperature that dissolves during urine production over 4–6 h, allowing mitomycin to act at the tumor site. A total of 74 patients were enrolled in the OLYMPUS trial, with 71/74 receiving at least one dose of UGN-101, while 61 completed the 6-weekly instillation protocol. Of the 71 patients who received induction therapy, 42 achieved a complete response, and 41/42 initiated follow-up. Of these 41 patients, 56% remained in complete response after one year with or without maintenance treatment. The most common adverse event related to UGN-101 instillation was ureteric stenosis, while urinary tract infection, hematuria, and flank pain were also related to drug administration and/or the procedure. However, there was no statistically significant difference in the mean eGFR change before, during, or after treatment. Therefore, instillation of UGN-101 appears to be effective in the treatment of low-risk localized UTUC, with a low rate of adverse events [33].

## 4. The Genomic Profile of Primary UTUC

Despite sharing a common histological origin, UTUC and UCB have distinct molecular and genetic features. Based on the Cancer Genome Atlas (TGCA) next-generation sequencing (NGS) data and following consensus between several major research groups, the molecular landscape of UCB can be classified into six subtypes: luminal papillary (24%), luminal non-specified (8%), luminal unstable (15%), stroma-rich (15%), basal/squamous (35%), and neuroendocrine-like (3%) [34,35]. However, since UTUC has a much lower incidence, fewer studies have focused on genomic and transcriptomic features of UTUC [36,37,38,39,40,41,42,43]. Combining and comparing NGS mutational and expression data from UCB and UTUC supports a luminal papillary contexture of the latter [44]. The most frequently mutated genes in UTUC are FGFR3, KMT2D, KMT2A, and TP53, but there are also mutations involving other oncogenes or tumor suppressor genes, including HRAS, NRAS, KRAS, ARID1A, PIK3CA, and CDKN2A, the frequency of which does not differ between UTUC and UCB.

**Figure 1:**
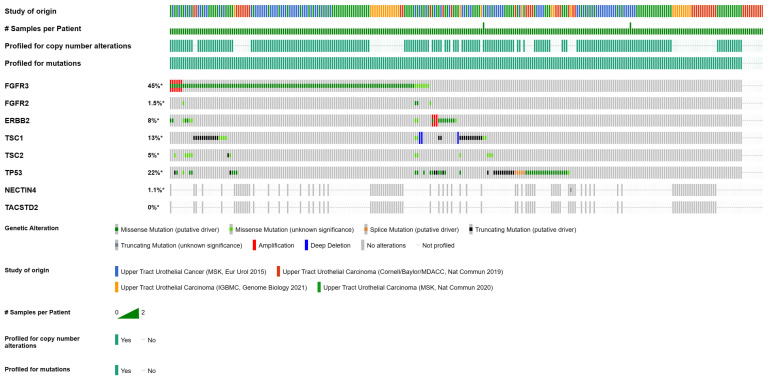
Frequency of common and targetable gene alterations in UTUC. *: percent of altered of queried samples. #: number of samples.

**Figure 2:**
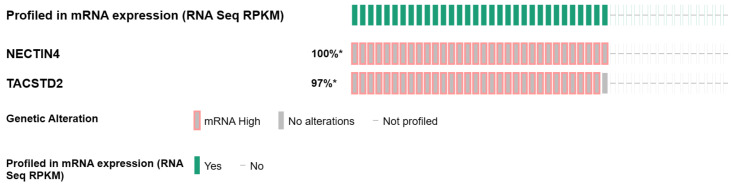
mRNA expression of the ADC targets NECTIN4 and TROP2 in UTUC from the Cornell/Baylor/MDACC Nat Commun 2019 study [44]. *: percent of altered of queried samples.

From a targetable alterations perspective, FGFR3 represents the most frequently mutated and/or amplified gene in UTUC, in 40% to 80% of sporadic UTUC, both high- and low-grade [36,37,44]. FGFR2 is much less frequently mutated (1.5%), with fusion status not included in these analyses (Figure 1). Oncogenic addiction to other altered signaling pathways, including human epidermal growth factor receptor 2 (HER2)- and mammalian target of rapamycin (mTOR), is not uncommon in UTUC, with ERBB2-activating mutations and/or amplifications detectable in 8% of cases and TSC1/TSC2-truncating mutations and/or deletions in up to 18% (Figure 1). These vulnerabilities are worth targeting; monoclonal antibodies (trastuzumab), tyrosine kinase inhibitors (everolimus), and even ADCs (RC48-ADC) have yielded promising results in UTUC [45,46,47]. TP53 mutations, although not yet directly targetable, are reported in up to 30% of UTUC cases, particularly in high-grade tumors with increased genomic instability, and are associated with more aggressive disease and poorer clinical outcomes (Figure 1). The mutation frequencies of the ADC targets nectin-4, encoded by the NECTIN4 gene, and TROP2, the gene product of TACSTD2, are close to 0% in primary UTUC tumors (Figure 1). In line with these findings, their mRNA expression status is high in 97–100% of tested tumors, confirming their universal presence and rational for targeting (Figure 2).

Interestingly, one-quarter of patients with UTUC harbor mutations of the ZFP36 family of tumor suppressor genes, especially ZFP36L1. ZFP36L1 is a zinc-finger RNA-binding protein that regulates several cytoplasmic AU-rich element (ARE)-containing mRNA transcripts by favoring their poly (A) tail removal or deadenylation, leading to the attenuation of protein synthesis [48]. Furthermore, analysis of ZFP36L1 knockdown has revealed disruption of the cell-to-cell junctions and a clear change to spindle-shaped morphology in the cells; this was associated with a loss of E-cadherin expression, consistent with the epithelial–mesenchymal transition (EMT). However, these mutations of ZFP36-family genes were not associated with clinicopathological tumor features and patient overall survival [48].

DNA-methylation changes are also present in UTUC tumors and may have an impact on patient outcomes. Methylome-wide analysis segregated UTUC into two DNA methylation-based epi-clusters: EpiC-C1, which presented a frequently hypermethylated profile and was redesignated as EpiC-high; and EpiC-C2 which presented a hypomethylated profile compared to EpiC-C1 and was therefore redesignated as EpiC-low [48]. Furthermore, these clusters were associated with somatic gene mutations. The EpiC-high cluster was enriched in SWI/SNF gene somatic mutations, more often associated with a muscle-invasive type of UTUC (69%) resulting in a shorter overall survival, while EpiC-low was enriched in FGFR3 mutations associated with non-MI disease (92%) [48].

UTUC may also occur as a result of heritable Lynch syndrome, an autosomal dominant disorder characterized by a high predisposition to multiple primary malignancies and an early age of onset [49]. Lynch-related UTUCs harbor germline mutations in one of four DNA mismatch repair genes (MMR), hMLH1, hMSH2, hMSH6, and hPMS2, or mutations in the hEPCAM gene which result in the silencing of hMSH2. The loss of function in the MMR system results in microsatellite instability (MSI) throughout the genome. Among these MMR mutations, different studies have concluded that patients who carry hMSH2 mutations present a higher risk of developing UTUC [50,51,52]. However, a lower but positive mRNA expression of MMR genes can be seen in patients with sporadic UTUC, resulting in a low total mutational burden [44].

The recent classification of UTUC into five mutational subtypes—the hypermutated, the TP53/MDM2-mutated, the RAS-mutated, the FGFR3-mutated, and the triple-negative—could serve as an additional tool for the better diagnosis and management of UTUC [53]. The first subtype is associated with MMR mutations that are present in Lynch syndrome. The TP53/MDM2-mutated subtype has the most aggressive clinical course, while the FGFR3-mutated subtype is associated with low-grade histology tumors and an increased survival rate. The RAS-mutated subtype is associated with high-grade tumors and squamous cell differentiation, while the triple-negative subtype has a similar prognosis to the TP53/MDM2 mutated subtype [53].

## 5. The Immune Microenvironment of Primary UTUC

Tumor microenvironment, particularly immune cell infiltration, may play a key role in the host’s antitumoral response and patients’ clinical outcomes. Sporadic UTUC exhibits a high frequency of FGFR3 mutations, and recent studies suggest that these tumors are CD8 T-cell depleted [44,54]. The majority of UTUC tumors have downregulated T-cell-related (CD8A, CCL2, CCL3, CCL4, CXCL9, CXCL10) and INFG signaling genes. More importantly, there is an association between activated FGFR3 signaling and immune gene expression in UTUC tumors, whereby T-cell-depleted clusters demonstrate a higher expression of FGFR3. Conversely, several IFNG response genes such as BST2, MX2, IRF9, and GBP2 are upregulated after FGFR3 knockdown or after pharmacological treatment with FGFR3 inhibitors such as erdafitinib [44]. This upregulation of FGFR3 signaling correlates with the PPARG gene signatures resulting in the suppression of proinflammatory cytokine signaling [44,55].

In addition to somatic gene alterations, the epigenetic landscape of UTUC may also determine the T-cell phenotype of these tumors. Tumors with an EpiC-low methylation profile harbor FGFR3 mutations and can be characterized as “immune cold”, while those with an EpiC-high methylation profile harbor mutations of the SWI/SNF pathway and are associated with a higher level of tumor infiltrating lymphocytes (TILs) [48].

The immune checkpoint PD-L1 is one of the regulators of immune response to tumor cells, and the PD-1/PD-L1 pathway has an immunosuppressive effect that helps promote cancer development. Assessment of PD-L1 expression might play a role in the prognostication of UTUC, since immunohistochemical overexpression above levels of 20–25% have been associated with a poor prognosis [56,57]. Another immune checkpoint, CTLA-4, is also being investigated [58].

## 6. Management of Locally Advanced and Metastatic UTUC

### 6.1. High-Risk Localized UTUC

Patients with high-risk localized disease should undergo RNU with bladder cuff removal and lymphadenectomy. Administration of adjuvant platinum-based chemotherapy is now recommended in these patients instead of surgery alone (evidence level I) [59,60]. The POUT trial, a multi-center randomized control trial that included 261 patients, demonstrated a significant improvement in disease-free survival (DFS) at a median follow-up of 48.1 months in patients receiving adjuvant platinum-gemcitabine combination chemotherapy beginning within 90 days following RNU [60,61].

Retrospective RNU studies have revealed that a significant proportion of patients, including 20% with pT2 disease, 35% pT3-T4 disease, and 10% with pathologically positive lymph nodes at the time of surgery, could benefit from neoadjuvant therapy (NAC) [62]. Although cisplatin eligibility requires adequate renal function, only one-third of patients have an eGFR > 60 at the time of diagnosis [63]. After RNU, eligibility for platinum-based chemotherapy further decreases to 15% and 50% for a threshold of eGFR > 60 and >45, respectively. Small NAC studies have shown promising pathological downstaging and complete response rates, as well as lower disease recurrence and mortality rates compared to RNU alone [50,51,52,53,54,55,56]. This was indeed confirmed prospectively in a phase-II study of neoadjuvant split-dose gemcitabine–cisplatin resulting in high pathologic response rates (63%) that further translated into prolonged 2- and 5-year OS rates (93% and 79%, respectively) [60]. No randomized studies of NAC have been published yet for UTUC.

In addition to platinum-based chemotherapy, the testing of immune checkpoint inhibitors (ICIs) has gained significant interest for the treatment of high-grade localized disease in both adjuvant and neoadjuvant settings. The PURE-01 study [64] reported prominent results of neoadjuvant pembrolizumab in muscle-invasive bladder cancer (MIBC) [61], whereas the PURE-02 study focused only on UTUC patients with high-risk features according to EAU guidelines [59,65]. In this study, 10 patients with high-risk nonmetastatic UTUC were enrolled. Patients received three courses of 200 mg intravenous pembrolizumab every 3 weeks and then underwent RNU within 14 days from the last dose. After the completion of treatment, one patient presented a complete clinical and radiographic response while the remaining patients were characterized as uncertain responders or overt non-responders. Two (20%) displayed disease progression and received additional cisplatin-based chemotherapy, prior to RNU. Overall, pembrolizumab was deemed inadequate as a single-agent neoadjuvant treatment for high-risk UTUC. The differences observed between the PURE-01 and PURE-02 trials concerning the efficacy of pembrolizumab in UCB and UTUC, respectively, may be at least partially explained by their distinct genomic and immunological features.

Several neoadjuvant single- or dual-agent immunotherapy and chemo-immunotherapy approaches have been tested in the neoadjuvant setting [66]. However, response rates have never reached those of cisplatin-based chemotherapy at the cost of significant adverse events, particularly from the dual ICI combinations, while long-term OS data and predictive markers are lacking.

The role of adjuvant immunotherapy with ICIs has also been studied. However, these trials included a limited pool of patients with UTUC. The IMvigor 010 study enrolled 809 high-risk UC patients who were randomized between adjuvant atelizumab and placebo. Only 7% of the patients receiving atezolizumab and 6% of the placebo arm had UTUC. After 19.4 months, there was no difference in median disease-free survival (DFS) between the two arms of the study [67]. The Checkmate 274 study enrolled 709 patients, with 27% of them diagnosed with UTUC, in order to study the adjuvant administration of nivolumab. This was a positive study leading to FDA approval of the drug, as patients receiving nivolumab presented a median DFS of 20.8 months whereas the placebo-treated group had a median DFS of 10.8 months [68].

### 6.2. Systemic Therapies for Metastatic UTUC

Cisplatin-based chemotherapy, including MVAC (methotrexate, vinblastine, doxorubicin, and cisplatin), or GC (gemcitabine and cisplatin), remains the standard of care for the front-line treatment of metastatic platinum-eligible patients [1]. RNU could be performed for palliative purposes and might offer a small benefit in the oligometastatic setting [1]. Analyses from three RCTs demonstrated that there are no significant differences in PFS and OS with respect to the location of the primary tumor in the lower or upper urinary tract [69]. However, approximately two-thirds of UC patients are not platinum-eligible, due to impaired performance status or comorbidities, and alternative chemotherapy regimens are less effective [70].

The addition of ICIs to the clinical armamentarium of systemic therapies for metastatic UC has provided a viable first-line option for platinum-ineligible patients and a life-prolonging approach compared to prior standard chemotherapeutic agents in the platinum-resistant setting. Atezolizumab and pembrolizumab were approved for PD-L1-positive patients with metastatic UTUC who are ineligible for platinum-based regiments based on promising results from IMvigor-210 and KEYNOTE-052 studies [71,72]. In the IMvigor-210 study, atezolizumab was associated with a median overall survival (OS) benefit of 15.9 months in cisplatin-ineligible patients with metastatic UC, one third of whom had UTUC disease [71]. In the KEYNOTE-052 study, pembrolizumab resulted in an objective response rate (ORR) of 22% in 69 patients (19% of all patients in the trial), with metastatic UTUC who were cisplatin-ineligible. This study also demonstrated an association between the expression levels of PD-L1 and the response to treatment, with a PD-L1 expression of at least 10% correlating with higher response rates [72].

Addition of atezolizumab and pembrolizumab to front-line platinum-based chemotherapy for patients with metastatic UTUC was investigated in two RCTs: the IMvigor-130 and KEYNOTE-361 trials [73,74]. However, the combination of ICI with platinum-based chemotherapy did not result in a significant improvement in OS compared to chemotherapy alone.

Only 10% of patients with metastatic UTUC undergoing first-line platinum-based chemotherapy will experience long-term remission, while the rest will exhibit disease progression [75]. ICIs including pembrolizumab, atezolizumab, avelumab, nivolumab, and durvalumab are approved as a second-line therapy for platinum-resistant disease [12]. Pembrolizumab has been shown to decrease the risk of death by almost 50% in those with UTUC according to findings from the KEYNOTE-045 trial, while patients receiving pembrolizumab demonstrated an ORR of 21.1% compared to those receiving second-line chemotherapy with an ORR of 11.4% [76]. Atezolizumab was initially reported to have a durable activity associated with PD-L1 expression on immune cells in patients with metastatic UC, with a reported ORR of 26% in a phase-II setting; however, the respective phase-III trial, IMvigor-211, did not confirm OS prolongation compared to chemotherapy [77]. A viable first-line maintenance strategy in platinum-responders based on findings from the JAVELIN Bladder 100 study is monotherapy with avelumab [78]. In this study, which included approximately one third of patients (30.3%) with upper tract disease, avelumab compared to the best supportive care significantly prolonged PFS and 1-year OS in PD-L1-positive patients, as well as in the overall study population [78].

The molecular profiling and subtyping of UC has offered several opportunities for targeting specific patient groups with distinct features. Despite the plethora of molecular alterations in UTUC, not all constitute therapeutic targets as of yet. One preselected group of patients with FGFR alterations was treated with erdafitinib, a tyrosine kinase FGFR1–4 inhibitor, after progression to platinum-based chemotherapy and/or ICIs [79]. Erdafitinib demonstrated significant activity, translating into a 40% ORR, median PFS of 5.5 months, and median OS of 13.8 months in those 99 patients [79].

Nectin-4 is highly expressed in UC and is targetable with a novel antibody–drug conjugate, enfortumab vedotin (EV). EV was highly active in mUC patients previously treated with platinum chemotherapy and ICIs, showing a high ORR of 44% with a median duration of 7.6 months in responders [80]. These findings were confirmed in a phase-III EV-301 trial, wherein EV was superior to standard chemotherapy with respect to median PFS (5.5 vs. 3.7 months) and OS (12.9 vs. 9.7 months) [81]. In cisplatin-ineligible patients previously treated with ICI (EV-201 phase-II trial), EV demonstrated an ORR of 52% [82]. An even more impressive ORR of 73% was shown when EV was combined in the phase-Ib/II EV-103 study with pembrolizumab in previously untreated advanced UC patients with a long median DOR and OS exceeding 2 years, with phase-III investigation (EV-302) ongoing [83]. In this trial, one third of patients (15/45) had upper tract disease [83].

A second ADC which is gaining ground as a later-line option for patients with mUC is sacituzumab govitecan (SC). SC was tested in Cohort I of a multi-cohort, open-label registration study in the post-platinum, post-ICI setting with two thirds of patients displaying liver metastases; SC showed a remarkable ORR of 27% and a median PFS and OS of 5.4 and 10.9 months, respectively [84]. An overview of prospective studies on various systemic therapies in UC that included UTUC patients are described in Table 1.

Due to the rarity of UTUC, most of our knowledge derives from studies that have enrolled both patients with UCB and UTUC; in all of these studies, UTUC represented a small proportion of the population. Therefore, the efficacy of current and future therapies in this rare type of tumor necessitates dedicated studies in UTUC patients exclusively.

## 7. Predictive Markers of Therapeutic Response

Clinical and radiographic responses induced by platinum chemotherapy and immune checkpoint inhibitors in locally advanced and metastatic UC are observed in a proportion of patients, with varying degrees of clinical benefit and duration. Consequently, there is an unmet need for developing pre-treatment biomarkers to better select patients who are more likely to respond. Contrary to activating mutations and amplifications in FGFR-pathway genes which successfully predict sensitivity to FGFR inhibitors, such as erdafitinib [79] and infigratinib [85], immunohistochemical assessment of PD-L1 expression remains problematic as a surrogate of ICI activity, particularly due to discordances among the different assays and cut-off points used for different anti-PD-1/PD-L1 monoclonal antibodies [86]. Thus, the clinical utility of PD-L1 immunohistochemistry is currently limited to predicting which cisplatin-ineligible patients should not receive frontline ICI [87]. A newer study retrospectively evaluated the predictive value of PD-L1 gene (CD274) amplifications and losses for ICI response and resistance, respectively; however, no definitive conclusions could be drawn due to its retrospective design and small size [88]. Additionally, despite the segregation of UC into molecular subtypes by several groups and a consensus on molecular taxonomy [89], there is no clear association between the subtypes and the responses to different therapies. One exception to this is the basal subtype which is highly responsive to platinum-based chemotherapy [90], while luminal papillary subtype is resistant to ICIs [79]. The occurrence of inactivating mutations in DNA repair genes has been associated with the response to both platinum chemotherapy and ICIs in retrospective studies [91,92,93]. A high tumor mutational burden (TMB) (≥9.65 mutations/megabase) emerged as a favorable predictor of response to atezolizumab in various UC cohorts in the IMvigor 210 and 211 studies [71,77,94]. A newer tool to predict the benefit from adjuvant ICI treatment with atezolizumab is the detection of positive circulating tumor DNA (ctDNA) [95].

While not all of the above predictive biomarkers are selective for UTUC-only disease, they have laid the groundwork for ongoing and future biomarker research. The B7 and CD28 families are crucial components of the immune checkpoint system, regulating immune responses through the activation and inhibition of co-stimulatory molecules [96,97]. The discovery of five new B7 family ligands (B7-H3 to -H7) has expanded our understanding. The presence of the co-inhibitory molecule B7 homolog 4 (B7-H4) in cancer cells may contribute to tumor progression by inhibiting T-cell proliferation and cytokine production within the tumor microenvironment [96,97]. Hence, B7-H4 plays a significant role in the theory of “immune escape” in tumors. Immunohistochemical expression of B7-H4 was recently studied in patients with primary UTUC who underwent RNU [98]. The high expression of B7-H4 was associated with later lymph node recurrence or distal metastases and predicted poorer treatment responses to chemotherapy [98]. Among patients with tumors harboring a high B7-H4 expression, the concurrent abundance of CD8 and T-cell intracellular antigen 1 (TIA-1), a marker of activated CD8, resulted in better treatment responses [98].

## 8. Conclusions

As our knowledge of the underlying biology and molecular vulnerabilities of UTUC expands, encouraging results from recent and ongoing prospective studies in this hard-to-treat patient population are paving the way for novel combinations of targeted therapies (Table 2). Most importantly, these studies have highlighted the unmet need to identify and validate robust biomarkers to guide treatment selection within a growing number of active pharmacologic agents.

## Figures and Tables

**Table 1:** Studies of systemic therapies in high-grade localized and metastatic UTUC.

Trial	Drug	Line	Total Patients/UTUC Patients	Main Outcomes
**High-grade localized disease**				
*PURE-02* [62]	Pembrolizumab	Neoadjuvant	10 patients with UTUC	-One (14.3%) patient achieved a clinical complete response -Two (20%) had disease progression -Seven patients underwent RNU: one (14.3%) achieved a ypT1N0 response
*POUT* [57]	Cisplatin or carboplatin + Gemcitabine	Adjuvant	261 patients with UTUC	3 y DFS 71% vs. 46%
*IMvigor 010* [64]	Atezolizumab	Adjuvant	809/54	median DFS 19.4 mos
*Checkmate 274* [65]	Nivolumab	Adjuvant	709/149	median DFS 20.8 mos
***Advanced/Metastatic Disease***				
*IMvigor 210* [71]	Atezolizumab		119/33	ORR 23%
*KEYNOTE 052* [72]	Pembrolizumab		370/69	ORR 24%
*IMvigor 130* [73]	Atezolizumab + platinum-based chemotherapy(A) vs. atezolizumab (B) vs. platinum-based chemotherapy (C)		1213/312	-Group A median PFS: 8.2 mos, median OS 16 mos -Group B median OS: 15.7 mos -Group C median PFS: 6.3 mos, median OS 13.4 mos
*KEYNOTE 361* [74]	Cisplatin or carboplatin + gemcitabine + pembrolizumab vs. pembrolizumab vs. cisplatin or carboplatin + gemcitabine		1010/211	median OS: 17 mos (pembro + chemo) vs. 14.3 mos (chemo) -median PFS: 8.3 mos (pembro + chemo) vs. 7.1 mos (chemo)
*KEYNOTE-045* [76]	Pembrolizumab vs. paclitaxel or docetaxel or vinflunine		748/75	-median OS: 10.3 vs. 7.4 mos -median PFS: 2.1 vs. 3.3 mos
*IMvigor 211* [77]	Atezolizumab vs. paclitaxel or docetaxel or vinflunine		931/236	median OS: 11.1 vs. 10.6 mos
*JAVELIN-100* [78]	Avelumab vs. BSC		700/187	median OS: 21.4 vs. 14.3 mos
*EV-201* [80]	Enfortumab vedotin		125/44	ORR: 44%, median DOR: 7.6 mos
*EV-301* [81]	Enfortumab vedotin vs. chemo		608/205	median OS: 12.8 vs. 8.9 mos median PFS: 5.5 vs. 3.7 mos
*EV-201* [82]	Enfortumab vedotin (cisplatin-ineligible)		89/38	ORR: 52%
*EV-103* [83]	Enfortumab vedotin + pembrolizumab		45/15	ORR: 73.3% median DOR: 25.6 mos median OS: 26.1 mos

Abbreviations: BSC: best supportive care; OS: overall survival; PFS: progression-free survival; ORR: objective response rate; DFS: disease-free survival; mos: months; DOR: duration of response.

**Table 2:** Non-exhaustive list of ongoing clinical trials in UTUC in various treatment lines.

Trial ID	Study Design	Treatment Line	Intervention	Primary Endpoint
NCT04228042	Ib	Neoadjuvant	Infigratinib	AEs, ORR
NCT04628767	II/III	Neoadjuvant	aMVAC +/− durvalumab	pCR
NCT05160285	II	Neoadjuvant	Nivolumab	pCR
NCT05564416	II	Neoadjuvant	Erdafitinib +/− atezolizumab	pCR
NCT04871529	II	Neoadjuvant	Avelumab + carboplatin/gemcitabine	pCR
NCT03244384	III	Adjuvant	Pembrolizumab vs. observation	OS, DFS
NCT02567409	II	1st	Cisplatin/gemcitabine +/− besosertib (ATR inhibitor)	PFS
NCT05092958	III	Maintenance	Avelumab +/− cabozantinib	OS
NCT04678362	II	Maintenance	Avelumab + talazoparib (PARP inhibitor)	PFS
NCT03237780	II	1st or 2nd	Atezolizumab +/− eribulin	AEs, ORR
NCT05564416	III	2nd	Eribulin +/− gemcitabine vs. standard chemotherapy	OS
NCT02496208	I	2nd	Cabozantinib + nivolumab +/− ipilimumab	RP2D, AEs
NCT03513952	II	2nd	Atezolizumab + CYT107 (interleukin-7)	ORR
NCT04724018	I	3rd	Sacituzumab govitecan + enfortumab vedotin	MTD, DLT, ORR
NCT03606174	II	2nd or 3rd	Sitravatinib + ICI	ORR

Abbreviations: AEs: adverse events; ORR: objective response rate; pCR: pathologic complete response; aMVAC: accelerated methotrexate vinblastine adriamycin cisplatin; OS: overall survival; DFS: disease-free survival; PFS: progression-free survival; RP2D: recommended phase-II dose; MTD: maximal tolerated dose; DLT: dose-limiting toxicity; ICI: immune checkpoint inhibitor.

## Data Availability

Publicly available datasets were analyzed in this study. This data can be found here: https://www.cbioportal.org/study/summary?id=utuc_cornell_baylor_mdacc_2019%2Cutuc_mskcc_2015%2Cutuc_igbmc_2021%2Cutuc_msk_2019 (accessed on 18 April 2023).
